# Rewards and recognition for Canadian distributed medical education preceptors: a qualitative analysis

**DOI:** 10.12688/mep.19152.2

**Published:** 2022-06-17

**Authors:** Amanda Bell, Aaron Johnston, Edward Makwarimba, Rebecca Malhi

**Affiliations:** 1 Niagara Regional Campus, Michael G. DeGroote School of Medicine, McMaster University, St. Catharines, Ontario, L2S 3A1, Canada; 2 Distributed Learning and Rural Initiatives, Cumming School of Medicine, University of Calgary, Calgary, Alberta, T2N 4Z6, Canada; 3 Office of Rural and Regional Health, Faculty of Medicine and Dentistry, University of Alberta, Edmonton, Alberta, T6G 1C9, Canada

**Keywords:** Distributed Medical Education, faculty recognition, preceptors, qualitative research

## Abstract

**Background**: Recognition of Distributed Medical Education (DME) preceptors by medical schools ensures that important community-based training opportunities remain available to learners. Yet the literature seldom explores what rewards are meaningful to this population of teachers. The goal of our national project was to provide guidance to medical schools about the financial remuneration and non-financial rewards that are most valued by DME preceptors.

**Methods**: In this qualitative study, we invited DME faculty members from all Canadian medical schools to participate in semi-structured interviews. Participants with a range of medical specialties, stages of career, and geographic locations were interviewed via Zoom videoconferencing. The sessions in English and French were audio-recorded and transcribed. We used line-by-line inductive coding and thematic analysis to examine participant talk about meaningful preceptor recognition.

**Results**: Fourteen participants from multiple provinces were interviewed. Results indicated that the DME faculty are a diverse group of people with diverse needs. Most of the interviewees appreciated the rewards and recognition provided by their medical schools but felt that there are areas for improvement. Recognition is not necessarily monetary and should be tailored to the needs and the values of the recipient. Other themes included: benefits and challenges of being a preceptor, current institutional structures and supports, and the impact of the pandemic on preceptors.

**Conclusions**: The interviews highlighted the importance placed by preceptors on personal rewards and a wide variety of forms of recognition. Based on the findings, we suggest specific steps that medical schools can take to support, engage, and recognize DME faculty.

## Introduction

Distributed medical education (DME) is a meaningful pillar in most Canadian medical schools ^1^. The relationship between preceptors and learners, particularly in rural settings, is one of engagement and trust. Importantly, this relationship must be anchored in the understanding of the individual student and formative in the development of the learner as a future physician ^2^. International studies show that rural community-based medical education facilitates mutually beneficial relationships among students, clinicians, patients, and community stakeholders ^3^.

Potential advantages to placing medical learners in community-based training sites include stimulating graduates’ interest in rural careers, developing their clinical competency and professional identity, and improving patient access to rural health care services ^3^. There are advantages for preceptors too, as studies indicate that they are motivated by a variety of intrinsic and extrinsic factors ^4^. However, faculty in distributed settings must balance their teaching commitments while simultaneously providing clinical care to their community. This can result in reduced income as the financial stipends associated with teaching are generally lower than clinical earnings.

Appropriate remuneration and recognition of DME faculty is critical to ensuring these important training opportunities remain available to medical learners. However, there is little information in the medical education literature that explores what is meaningful to this population of preceptors.

The current study is part of a larger research project that we titled ‘Currencies of Recognition.’ We chose this term to acknowledge the multiple ways of recognizing faculty contributions, and to emphasize that effective recognition will hold value for both the giver and the receiver. Results from the quantitative exploratory phase were previously reported by Johnston *et al.*, (2022), and the qualitative study builds upon this work ^5^. The overarching goal of the Currencies of Recognition project is to provide guidance to medical schools about the financial remuneration and non-financial rewards that are most attractive, effective, and practical in recognizing community preceptors for their contributions to medical education. Meaningful recognition and remuneration of faculty will help medical schools to recruit, support, engage, and retain DME faculty for many years to come.

## Methods

### Ethics

The current study was reviewed and approved by the University of Calgary Conjoint Health Research Ethics Board (CCHREB) – Ethics ID: REB19–1132. Participants were sent electronic copies of the informed consent form in advance of the interviews, and they provided verbal consent prior to the interview. The CCHREB required that data be kept on a secure local server only for a period of seven years after completion of the study and then deleted.

### Study design

The Currencies of Recognition project originated at the Association of Faculties of Medicine of Canada (AFMC) DME meeting during the Canadian Conference on Medical Education (CCME) in May 2019. The major focus of the research was DME faculty engagement. Project members were all involved with DME, including individuals in leadership roles, research roles and administrative roles. The team included people from a mix of settings: rural and urban locations, as well as both French language and English language medical schools. Members of the group met every one to two months over Zoom throughout the study. All members of the group had previous research experience and specific members of the group had expertise in each of quantitative research, qualitative research, and statistics.

We designed the project with two phases: an initial national quantitative survey and a follow-up interview-based qualitative phase. Some of the results from the survey informed the design of the second part of the project. This paper primarily reports on the findings from the qualitative interview phase.

The initial study recruitment was conducted through DME leaders at all Canadian medical schools. A bilingual introductory letter that included a link to an online survey was electronically emailed to DME leaders at each institution. The contacts at each school were the identified DME leads who make up the membership of the national AFMC-DME group. The medical school contacts (members of the national AFMC-DME group) were requested to forward the letter and survey to their eligible DME faculty. To obtain national representation of participants, all materials were translated into French by a professional translator at the University of Calgary, Canada.

The survey collected data related to the current forms of recognition provided to DME preceptors, and the value preceptors place upon each recognition type. Survey data was analyzed statistically and the results of the quantitative phase of this project have been reported elsewhere ^5^. Many respondents also wrote in the free text fields provided on the survey. These written comments were exported from the survey instrument into Microsoft Word tables. Comments originally in French were translated into English by a professional translator in the Linguistics department at the University of Calgary. The data were then analyzed qualitatively in NVIVO 12 using a structured, inductive approach based on thematic analysis ^6^. Two members of the research team with qualitative expertise (RM and AB) performed independent analyses. We used the themes that emerged from the analysis of the free text fields to inform the interview guide for the semi-structured interviews.

### Data collection and analysis

For the qualitative phase, we contacted all survey respondents who had expressed a willingness to participate in a longer follow-up interview. From the list of possible interview candidates, we sent invitations to a selected number of participants with a range of medical specialties, stages of career, and geographic locations. Informed consent was obtained from all interview participants.

Interview data was collected between 5 ^th^ January and 5 ^th^ February 2021. RM and AB conducted semi-structured interviews in English via Zoom videoconferencing. RM had no pre-existing relationships with participants, but AB had a leadership role with some of the DME faculty. To avoid biasing the data, the researchers interviewed only participants who were unknown to them.

One interview was conducted in French by a research assistant. Each interview lasted approximately 45 minutes. The researchers focused on obtaining information about the intrinsic and extrinsic rewards of being a preceptor, the diversity of preceptor recognition currently offered by the participant’s institution, the value placed on these by preceptors, and suggestions for forms of recognition that would be appreciated but are not currently offered. We used an interview guide ^7^ to keep the conversations on track. Interviews were audio-recorded and transcribed verbatim by a transcription company. The French language interview was transcribed in French and then translated to English by a professional translation service. All transcripts were de-identified before analysis.

Interviews were analyzed as they were completed, and the interview/analysis cycle continued until saturation of themes was reached within the dataset. Two team members (AB and RM) analyzed all the interview transcripts with the aid of NVIVO v12 qualitative software. A third team member (EM) independently analyzed the transcripts in Microsoft Word.

During the initial analysis, we used line-by-line inductive coding, created memos, and provided preliminary interpretations. Comments with multiple concepts could be assigned to more than one code; a process of constant comparison between codes was used to systematically categorize, compare, and evaluate the data. In order to ensure the trustworthiness and credibility of the analysis, after the first iteration of coding, RM and AB assessed whether they were achieving consensus with the coding. Thereafter, they met regularly to discuss memos, additional codes, and emerging themes. EM reviewed our analyses and provided feedback. We used thematic analysis ^6^ as an appropriate methodological framework to examine participant talk about how preceptors are recognized.

## Results

Fourteen interviewees took part in the qualitative phase of the project. Participants were engaged in medical practice in multiple provinces including British Columbia, Alberta, Saskatchewan, Manitoba, Ontario, Quebec, and New Brunswick. They were primarily family physicians, but we also had participation from physicians practising Emergency Medicine, Internal Medicine, Pediatrics, Geriatrics, Obstetrics, Psychiatry, Acute Care, and Anesthesia. They ranged in experience from four years of teaching to greater than thirty years of teaching. All participants held faculty roles at their home medical schools, and many also held leadership positions at universities, provincial health organizations, hospitals, and communities.

Themes that emerged from the interviews included: the benefits and challenges of being a preceptor, institutional supports, rewards, and recognition that are currently offered, rewards and recognition that is valued or suggested, impact of the pandemic on preceptor recognition and satisfaction of preceptors. Illustrative quotes are provided for each theme.

### Benefits of being a preceptor

Most of the respondents described various intangible, intrinsic rewards associated with being a preceptor. For several preceptors, a love of teaching had naturally drawn them into the role. They also expressed satisfaction with the variety of tasks they handled. Interacting with learners was viewed by respondents as being personally fulfilling. Preceptors enjoyed seeing their learners succeed as they grew in skills and confidence. One respondent said, “…And that’s definitely very emotional at the graduation, you know, especially somebody who had a hard time and kind of overcame certain obstacles. It’s very rewarding to see that – you know how far they made it and definitely recognizing the little contribution that we had in that” (AB5). Several preceptors also noted that having enthusiastic medical students was fun: “…I think by having a learner there it helps at least to reinvigorate your interest in just learning medicine…You know, when you teach a learner something for the first time, seeing their excitement kind of reminds you that it used to be exciting for us too” (AB6). Some interviewees expressed pride in furthering the medical profession: “It’s giving back. So, it’s providing the same service that I received when I was getting trained” (AB4).

There were also professional benefits, including access to opportunities for mentorship, leadership, and faculty development. Many preceptors stressed that learning was a continuous and two-way process. Supervising learners gave DME faculty the opportunity to maintain and update their own skills, which ultimately benefited their patients. One respondent stated, “I find it really helps cement my knowledge and also I learn from the learners. It keeps me current. It’s also great learning from different learner perspectives, people who have had different backgrounds or may have grown up or experienced different settings. So, I find it helps shape my practice” (RM7). Preceptors often developed lasting relationships with their learners, and in some cases, the learners become colleagues when they returned to establish a practice in the community.

Another professional benefit included networking with other DME faculty: “Again, just that community of preceptors that you get across the province that you wouldn’t get otherwise, is just really neat. If I wasn’t precepting, I wouldn’t, I wouldn’t have that extra community” (AB3). This community of DME faculty developed remarkably close bonds, even though they might be in geographically distant locations: “It doesn’t matter that I have anaesthesia training and my colleague doesn’t or my colleague is a surgeon or whatever. Like we all sort of recognise we have our strengths, and we listen to each other. It’s an extremely respectful level playing field” (RM1).

### Challenges of being a preceptor

Despite the intrinsic rewards that were associated with interacting with learners, some DME faculty stated that their efforts deserved additional compensation: “[Teaching] is a reward, but it’s – it can be tiring, and it takes a lot of time and effort. So, while I do have students that I teach, for example, when I have nurse practitioner students you don’t get paid, or you don’t get any financial remuneration for taking them. But we still have them because we enjoy teaching. So, there is aspects of that. But on the medical side, I think I would still want to be financially remunerated to have clerks, pre-clerks and residents” (AB4).

Time management was often the greatest challenge reported by DME faculty. Supervising learners means extra time that preceptors must spend on teaching and administration, sometimes leading to “…longer days when I have a learner” (AB1). Preceptors with a clinical practice often experienced a slowdown in clinic flow or had to decrease clinical hours, which in turn, affected their finances: “But you know all the time that I’ve put into teaching, if I saw patients, or if I did more clinical duties, I would make a much higher salary, but that’s not what I’m looking for, right?” (AB5). For some preceptors, being slowed down by students is not arduous because “…you get a little bit out of the deal too” (RM5). However, struggling learners added to the time challenge, and emotional burden. As one preceptor noted, “Especially with a learner that’s in difficulty; that takes up a lot of time and effort and energy, physical, mental” (AB5).

### Current institutional structures and supports

During the interviews, DME faculty said that they are, for the most part, satisfied with the current institutional structures. They did report minor irritations with some bureaucratic procedures, such as increased paperwork and administrative tasks associated with teaching and scheduling. Most preceptors feel supported by their medical schools, or do not identify needing support. DME faculty working in remote locations generally felt that they are not disadvantaged, compared to central campus faculty. However, they believed that their perspectives and challenges are not always considered. For example, several preceptors mentioned that the location and scheduling of meetings were not always convenient. Another noted that because the rural branch is so out-numbered by the central preceptors, they are not on an equal footing: “So, I am invited to meetings, and so we’ll sit around the table and there’s two rural people and twelve urban, and so your voice gets lost. I have sat in many meetings where the focus is purely one of the big (urban) centers, and it’s got nothing to do with me and I’ve driven all that way to go to this meeting” (AB1).

Distributed faculty have a substantial amount of autonomy and flexibility in shaping their programs: “…We can kind of mold the way that we want to do learning and teaching at our site, without having any overarching eyes on us, I guess. That might be not a good way to phrase it, but we can feel like we can really home-grow our training and our trainees in that way” (AB4). As noted earlier, there is a great sense of community and close relationships between DME preceptors. However, they can face challenges related to isolation and role overload. DME faculty are also more likely to struggle with the “administrative burden” (CP1) of supervising learners than preceptors at the central campus, especially since they have less administrative support.

### Rewards and recognition that are currently offered to DME faculty

DME faculty receive a variety of rewards and recognition from their medical schools. These offerings can be described in terms of the ‘clusters of recognition’ that we found in the survey results ^5^. DME faculty described being provided with *Tokens of Gratitude*—items that include plaques, certificates, and small gifts. Although some individuals appreciated these small rewards, other preceptors did not find them to be meaningful: “I’ve gotten like a plaque of some kind for some sort of award of recognition at one point. To be honest, don’t care so much about that. I’m not – I couldn’t tell you what it was even” (AB3). 

More highly valued were items that fell under the *Formal Institutional Recognition* category. These included awards for years of service, peer-nominated and student-initiated awards. Student-initiated recognition was particularly valued. Sometimes the awards were specifically for DME faculty. Preceptors also mentioned that their medical schools hosted formal recognition events where awards are presented (e.g., gala dinners). Some DME faculty noted that the awards process was flawed because many deserving preceptors did not get recognized: “If you have 15 people who are going above and beyond and just doing wonderful work and one of those 15 gets nominated, that’s lovely for that one person but then you’ve got 14 other people who are doing perhaps extraordinary things and is there any way to recognize that in some way?” (RM4).

Preceptors also mentioned that they received rewards that could be classified under the *Connections, Growth and Development* cluster of recognition. Items in this category were highly valued by DME faculty, and included Faculty Development training, conferences, and networking opportunities. Some preceptors also had access to institutional resources and amenities, and potential funding.

Finally, DME faculty did receive financial recognition, in the form of an honorarium or stipend. Most preceptors view this remuneration as a fair exchange for their services. One interviewee said, “So we should be compensated for the expertise that we’re providing in the education of the students” (RM3), and bluntly stated that without the stipend, he would not be a preceptor. On the other hand, another preceptor expressed ambivalence about receiving financial compensation at all: “I don’t really care about any of that stuff quite honestly…the work itself is its own reward. So I mean it’s baked into the work itself.” (RM5).

In general, the amount of the stipend was appreciated: “Yeah. So, remuneration is important, because as I said, it’s a lot of work to be a preceptor, regardless of how much you love and enjoy it. It’s nice to get that remuneration so that you can cover any expenses if need be. So that’s actually quite important, I guess, going forward” (AB4). However, the financial compensation was often not commensurate with the extra time and administrative burden associated with the learners.

### Rewards and recognition that are valued or suggested by DME faculty

Participants in this study were clear that forms of recognition that were personalized and genuine held the greatest value for them. However, many interviewees noted that communications from central departments or programs about their teaching—including awards—were often generic or not relationship-based. These messages unintentionally served to distance the faculty members from the university and from their roles as preceptors. One participant stated, “I don’t think they even know I exist, to be honest. I mean other than I’m a name on an email list” (RM1). This was a common sentiment shared by participants in response to mass communication from the university. To counteract this lack of personal approach, preceptors felt in-person visits were important and encouraged senior leadership to take the time to visit the distributed sites from time to time to best understand the setting, the needs, and the opportunities for medical education. DME faculty saw this as a very feasible way of recognizing the time and energy they put into teaching. One interviewee suggested, “Being more personable and more approachable and maybe having a relationship with your faculty members would be a good start and it wouldn’t cost anything other than time” (RM1).

As noted earlier, participants appreciated the financial compensation—honoraria and stipends—provided by medical schools. Although some respondents mentioned the need to increase remuneration, money was not the primary motivator for precepting. Respondents did suggest and highly value recognition that would help them save time or accomplish more academically. These rewards included increased administrative support, decreased paperwork, or ease of completing work such as evaluations. Some preceptors also requested increased support for research in the community.

DME faculty were eager for timely and specific feedback on their teaching as well as faculty development opportunities targeted to their needs and growth. They wanted to be asked about their experiences as preceptors and to be genuinely thanked for their work. They expressed a desire for a balance of teaching between early learners and more senior learners. There was no one-size-fits-all solution to the rewards valued by faculty. However, certain rewards were considered to be important by preceptors and were genuinely appreciated: recognition of years of service, awards that were locally created and chosen, and personal recognition in smaller ways of the efforts they are putting in. This sentiment was well summarized by one preceptor who stated, “You have to – not literally financially, but you make the deposits of trust, and then you can withdraw after you’ve built it up. And you can ask people to do extra.” (RM3).

### Impact of the COVID-19 pandemic on DME faculty recognition

Given the timing of the interviews with participants in this research project was during early 2021, the topic of the impact of the global COVID-19 pandemic on medical education came up frequently in discussions. Interestingly, faculty members described the pandemic as being a “great equalizer” between urban and distributed faculty and student experience in many ways. Events such as faculty development offerings and awards ceremonies were previously hosted in central urban locations and thus were less accessible to faculty teaching at distributed and rural sites. The transition of many of these offerings to online, virtual, and technology-facilitated events increased the access and ease of participation for rural preceptors. Faculty also noted that students no longer needed to go “back to the city for various extra bits of teaching” (AB3), which improved their continuity in their rural placement and “it’s brilliant for us” (AB3). The rapid changes in medical education delivery necessitated by the pandemic broke through barriers of distance and access that had existed for distributed medical education. One participant stated, “There have been more and more incentives for remote learning, which has grown rapidly during the last two years. Now with COVID, it has rocketed” (CP1).

### Satisfaction of DME faculty regarding recognition

Participants in this study were, overall, very satisfied with the support they received as DME preceptors. This could be a reflection of a bias in participant self-selection; preceptors who were disengaged, feeling unsupported, or frustrated with their experiences as distributed faculty may have chosen not to participate. The faculty who did participate in this study generally expressed feelings of being supported and that the university understood the sacrifice and commitment they were making in teaching medical trainees. Distributed faculty felt strongly connected to their regional sites and leadership and knew who to approach for help with challenging situations, particularly learners in difficulty. They also described the strong community feeling among rural preceptors as being an important source of information support: “Aside from the formal training there’s been people who’ve been in the role longer that say, ‘Hey, reach out any time if you have any issues.’” (RM7). The independent spirit and problem-solving attitude of preceptors away from urban centers and tertiary hospitals was also evident in the satisfaction they demonstrated with their position as distributed faculty. One preceptor said, “We just make things work, and if we need help, we’ll ask for it, but more often than not, we don’t need it and we just get the job done.” (RM2).

## Discussion

The interviews from this second phase of our project indicate that faculty in distributed medical education are generally satisfied with their work and the recognition they receive. Our findings are congruent with the findings in the first phase of the project. Both quantitative and qualitative results document the disparate value placed by different faculty on recognition within the clusters of formal institutional recognition, connections, growth and development and tokens of gratitude. In particular, the interviews corroborate the importance placed by preceptors on personal rewards and a wide variety of forms of recognition. Zelek and Goertzen (2018) presented a framework based on motivation theory that identifies many of the same intrinsic motivators and extrinsic rewards described in our results ^4^. Thus, our findings confirm prior empirical research.

The interviews reinforce the concept that DME faculty have an intrinsic love of teaching as previously reported in the literature ^4, 8, 9^, and appreciate the intangible rewards and community services that are associated with being a preceptor. There was a strong sense of community and identity within these preceptors. Faculty did recognize the time and effort required to fill the role of preceptor, particularly when students were having difficulties. The increasing role of technology and virtual offerings improved the experience of distributed faculty to participate in events and training, and worked as an equalizing factor among preceptors working in distributed sites.

### Recommendations

Results from this study indicate that faculties of medicine and individual medical campuses across the country can take specific steps immediately to support and recognize community-based preceptors who are working away from major academic sites. The findings contribute to further understanding of, and ways to increase, engagement of distributed medical faculty; these areas are currently noted in the literature as being complex ^4^.

A prominent theme in our findings was participants’ desire for personal connection. Medical schools should look for opportunities to satisfy this need, such as facilitating ways in which faculty can connect with students as individuals and with peers as part of a community of teachers in their own setting. Preceptors also appreciate personal connection with institutional leadership, so medical schools should consider virtual or in-person site visits by leaders, handwritten notes, personalized recognition for years of service, or individualized invitations. Connections with communities could also be strengthened. Distributed faculty have a strong sense of pride in their work and identity; their accomplishments could be highlighted to the institutional and local communities through awards, media coverage, and newsletters.

DME faculty also indicated that there are times where they require additional support. Institutional leadership could provide preceptors with in-kind services and opportunities through the existing faculty infrastructure. For example, assistance with the faculty appointment/promotion process or supplementary administrative support for research or paperwork related to teaching would be highly valued. A different kind of support is needed when learners or faculty are facing challenges. In such cases, providing practical assistance helps to acknowledge the burden of teaching, and facilitates a smooth and successful experience for both learner and faculty.

Financial remuneration is another area of complexity which should be examined by institutional leadership. It would be inaccurate for programs to assume that monetary motives are the sole drivers for the recruitment, retention, and satisfaction of preceptors. Given the current era of constrained budgets and cost-saving measures, medical schools have limited ability to provide additional compensation. Although the DME faculty we interviewed appreciate receiving extrinsic motivation like stipends and honoraria, being provided with opportunities to support their intrinsic motivation seemed to hold higher value. For example, fostering an environment where preceptors are given recognition by their peers and students could be achieved at little to no financial cost to the institution.

DME faculty are often unaware or misinformed about remuneration and resources available to central faculty, so programs must be transparent about any existing financial incentives. We also suggest that medical schools remain flexible in compensating DME faculty. Given that the value of a particular reward is dependent on the recipient, it would be prudent to offer preceptors diverse ‘currencies’ of recognition from the ‘clusters’ we identified in the first phase of our project ^5^. It should also be acknowledged that money is only one of the tokens of recognition offered to DME faculty, and may not fully compensate for any loss of clinical earnings they may experience when taking on learners.

The impact of the COVID-19 pandemic was an important theme in the results. Respondents highlighted the positive impact of the pandemic around distance learning and meeting technologies. Distributed medical education preceptors found it easier to participate in faculty development and participate in meetings and the academic process of the medical school. They also noted the positive impact on learner continuity: learners were able to stay in the community continuously and participate remotely in education, rather than travelling to a central site for these sessions.

At a higher policy and procedural level, there are actions that can serve to improve inclusion and recognition of distributed medical faculty within the institution. Access to resources that support the work of faculty, including administrative and research support, needs to be considered not only at the central sites where there tend to be larger programs, but how that is extended to distributed faculty. Recognition of the faculty role through awards, appointments and inclusion at decision-making tables needs to be viewed through a lens of the diversity of the faculty population. This diversity includes various geographic locations, access to health resources, and part-time as well as full-time positions. Academic funding models should be clear to those that are in the programs, but also to those who do not benefit from this funding. This would help to decrease the inaccurate belief that faculty in central departments are much more highly resourced or supported for their academic work.

### Limitations of study

While efforts were made to include a cross-Canadian range of participants, there were several provinces and territories that were not represented. Additionally, the participants were mostly family physicians. It is quite possible there are other priorities and unmet needs for physicians from other settings and in other specialties. Many of the physicians interviewed had previously held leadership roles. This subset of the sample might have more awareness of the academic system than people without leadership experience, and be more willing to engage and navigate the system. Finally, the physicians we interviewed were largely very satisfied with their role as teachers and the recognition they received. Targeting physicians who are not satisfied or who have left teaching due to feeling under-recognized might yield different priorities for currencies of recognition.

## Conclusion

This study highlights the motivations among DME faculty to teach and what forms of recognition are most important to them. Our results support previous research showing that the intrinsic rewards of teaching are highly influential ^4^. The importance of personal connections, the need for fair and transparent remuneration practices, and a range of types of recognition in order to broadly satisfy DME faculty are congruent with our previous research in this area ^5^. Our participants valued the connection to central campuses while also emphasizing the need for a degree of self-determination and input into decision making. To us, this indicates that recognition is not only about what the central campus gives to DME faculty but also how it takes feedback, suggestions and decisions. The take-home message for medical schools is that recognition is not just about showing appreciation for a job well done, but also about recognizing that DME faculty are in fact the experts in distributed medical education.

The results of this study can help distributed medical education units and their central medical campus to successfully engage with one another. There are many advantages to this beneficial relationship, including increased preceptor engagement, satisfaction, and retention, as well as excellent learning experiences for students. Future work in this area should include evaluating the impact of implementing an approach based on “currencies of recognition.” Theoretical frameworks and models of DME engagement should also be reviewed and extended.

## Data availability

### Underlying data

The underlying data for this study, including interview transcripts and NVIVO tables, contains potentially identifying information. Participants live and work in geographic areas with very few possible study subjects. The data is held on a secure server and only accessible by the research team as described in the data handling requirements approved by the University of Calgary Conjoint Health Research Ethics Board for this study (Ethics ID REB19–1132). The CCHREB approval for this study requires that the data be held on a secure local server for a period of seven years beyond the completion of the study. De-identified data can potentially be made available from the corresponding author upon reasonable request for the purpose of further research.

### Extended data

Open Science Framework: Rewards and recognition for Canadian distributed medical education preceptors: A qualitative analysis. (Johnston *et al.*, 2022)

https://doi.org/10.17605/OSF.IO/CTUWM

This project contains the following extended data:

● Interview guides (interview guide used in the study; available in French and English).

### Reporting guidelines

Open Science Framework: SRQR checklist for ‘Rewards and recognition for Canadian distributed medical education preceptors: a qualitative analysis’ (Johnston *et al.*, 2022)

https://doi.org/10.17605/OSF.IO/CTUWM

Data are available under the terms of the Creative Commons Zero “No rights reserved” data waiver (CC0 1.0 Public domain dedication).

## Software availability

The following software was used in the research study. For proprietary software a free alternative is also listed.

NVIVO version 12 is a proprietary qualitative data analysis software tool. A free alternative software tool is Taguette.

Zoom is a proprietary online video conferencing software platform that offers audio and video recording functions. A version of the Zoom software platform is available without charge.

Microsoft Word is a proprietary document creation and editing software platform. A free alternative software tool is Google Docs.
