# Supplementary material for: Rewards and recognition for Canadian distributed medical education preceptors: a qualitative analysis
Source: MedEdPublish (2016). 2022 Jun 17;12:36. Originally published 2022 May 16. [Version 2] doi: 10.12688/mep.19152.2 (PMC9370077; doi:10.12688/mep.19152.2)
Supplement: Peer review history

# Peer review: Reviewer response for version 2

**DOI:** 10.21956/mep.20553.r32168

**Published:** 2022-06-17

**Authors:** Andrew Hunt

**Affiliations:** 1 Memorial University of Newfoundland, St. John's, NL, Canada

Agree with the revisions.  No further comments to make.

Have any limitations of the research been acknowledged?

Yes

Is the work clearly and accurately presented and does it cite the current literature?

Yes

If applicable, is the statistical analysis and its interpretation appropriate?

Not applicable

Are all the source data underlying the results available to ensure full reproducibility?

Yes

Is the study design appropriate and is the work technically sound?

Yes

Are the conclusions drawn adequately supported by the results?

Yes

Are sufficient details of methods and analysis provided to allow replication by others?

Yes

Reviewer Expertise:

Distributed Medical Education, rural health care, rural health care leadership, rural physician recruitment and retention.

I confirm that I have read this submission and believe that I have an appropriate level of expertise to confirm that it is of an acceptable scientific standard.

# Peer review: Reviewer response for version 1

**DOI:** 10.21956/mep.20512.r32009

**Published:** 2022-06-06

**Authors:** Andrew Hunt

**Affiliations:** 1 Memorial University of Newfoundland, St. John's, NL, Canada

This is an excellent article pertaining to a very important component of medical education. The researchers are to be commended on such fine work. Distributed Medical Education (DME) is invaluable to both our future physicians and current preceptors. This article successfully highlights key concepts that contribute to DME preceptor satisfaction, and ultimately recruitment and retention. Recognition of DME faculty comes in many diverse forms, some with greater impact than others. It is important that DME faculty be appreciated for the great work they do in contributing to medical learner development. As challenges are many, we must focus on the positives to ensure integrity and longevity in DME. This article discusses these concepts in great detail.

 The authors have written an excellent article that is quite easy to follow from beginning to end. Each section's description is well-articulated in language that is clear and efficient. The study's goals and objectives are identified clearly, and the methods are described in sufficient detail to allow for a full understanding of study design and performance, including means of reproducibility. The results and conclusion describe well the findings and their applicability to DME across the country.

 Overall, this article helps to highlight the importance of DME preceptor recognition in Canada. Although the study population was somewhat diverse, its volume was relatively low and did not include representation from all provinces and territories in Canada. That being said, the authors acknowledged this limitation and provided reasonable statements regarding the influence of such a limitation on the study outcome.

 The two-part study referenced in this article is very important work in DME in Canada. This qualitative part two analysis is integral in nailing down the significance of DME preceptor recognition and should be shared widely.

 I have no reservations in recommending this article pass peer review. A very intriguing topic explored very well.

Have any limitations of the research been acknowledged?

Yes

Is the work clearly and accurately presented and does it cite the current literature?

Yes

If applicable, is the statistical analysis and its interpretation appropriate?

Not applicable

Are all the source data underlying the results available to ensure full reproducibility?

Yes

Is the study design appropriate and is the work technically sound?

Yes

Are the conclusions drawn adequately supported by the results?

Yes

Are sufficient details of methods and analysis provided to allow replication by others?

Yes

Reviewer Expertise:

Distributed Medical Education, rural health care, rural health care leadership, rural physician recruitment and retention.

I confirm that I have read this submission and believe that I have an appropriate level of expertise to confirm that it is of an acceptable scientific standard.

# Peer review: Reviewer response for version 1

**DOI:** 10.21956/mep.20512.r32012

**Published:** 2022-05-30

**Authors:** Roger Strasser

**Affiliations:** 1 TeHuataki Waiora School of Health, The University of Waikato, Hamilton, New Zealand; 2 NOSM University, Sudbury, Onatrio, Canada

The authors are to be congratulated on their success in undertaking this important research. As they state, Distributed Medical Education (DME) has become a feature of medical education across Canada and this study adds new knowledge about the experience and perspectives of the distributed clinical teachers, the majority of whom are in remote and rural community settings.

 Beginning with the article Title, each section (Abstract, Introduction, Methods, Results, Discussion and Conclusion) is written clearly with language that is understandable and accessible to readers of this online Journal. Specifically, the introduction provides the background and sets the scene for the study design that is presented in the Methods. The Results section is presented with subheadings that direct readers' attention to the key concepts distilled from the thematic analysis. In the Discussion, there is a brief summary of the findings and some mention of related research publications before moving on to the Recommendations that were foreshadowed in the Introduction. The conclusion reiterates the importance of this study and its potential to inform improvements in recognition and support provided by medical schools to DME faculty members.

 My only suggestion for improvement is that the authors replace the term "medical institutions" with either "medical education institutions" or "medical schools", because at first glance "medical institutions" could refer to a range of other organizations. 

 Essentially, I see this article as reporting rigorous research that adds new knowledge to the academic literature. I have no hesitation in recommending this article pass peer review.

Have any limitations of the research been acknowledged?

Yes

Is the work clearly and accurately presented and does it cite the current literature?

Yes

If applicable, is the statistical analysis and its interpretation appropriate?

Not applicable

Are all the source data underlying the results available to ensure full reproducibility?

Yes

Is the study design appropriate and is the work technically sound?

Yes

Are the conclusions drawn adequately supported by the results?

Yes

Are sufficient details of methods and analysis provided to allow replication by others?

Yes

Reviewer Expertise:

Rural health education and training, workforce and services research.

I confirm that I have read this submission and believe that I have an appropriate level of expertise to confirm that it is of an acceptable scientific standard.

# Response

**Published:** 2022-05-30

**Authors:** Aaron Johnston

**Affiliations:** Medicine University of Calgary, Canada

Thank you Dr. Strasser for your review and for your suggestion which we will incorporate into the updated version. I appreciate you taking the time to review this article. Aaron
